# The Initial Middle Eastern Experience With the Alterra Adaptive Pre-Stent: Single-Center Outcomes From Saudi Arabia

**DOI:** 10.1016/j.cjcpc.2025.09.003

**Published:** 2025-09-29

**Authors:** Mohamed Al Nasef, Bandar Al Shehri, Khalid Al Naji, Ahmed Al Zahrani, Merna Atiyah, Omar Abdulaziz Almohizy, Saria Mansour Ahmed, Khalid Al Najashi

**Affiliations:** a Pediatric Cardiology and Adult Congenital Heart Disease, Prince Sultan Cardiac Center, Riyadh, Kingdom of Saudi Arabia; b Suliman Al Habib Hospital, Khobar, Kingdom of Saudi Arabia; c King Hamd American Mission Hospital, Manama, Kingdom of Bahrain; d Adult Congenital Heart Disease, Prince Sultan Cardiac Center, Riyadh, Kingdom of Saudi Arabia

**Keywords:** Alterra prestent, percutaneous pulmonary valve implantation, Tetrology of Fallot

## Abstract

**Background:**

The Alterra Adaptive Pre-Stent, used with the Edwards SAPIEN 3 valve, offers a novel transcatheter option for patients with severely dilated right ventricular outflow tracts (RVOTs) previously unsuitable for conventional transcatheter pulmonary valve replacement (TPVR). No prior experience with this system has been reported from the Middle East.

**Methods:**

We report the first Middle Eastern experience with the Alterra Adaptive Pre-Stent from a high-volume congenital heart center. A retrospective, single-center analysis was performed at Prince Sultan Cardiac Center (Riyadh, Saudi Arabia). Between April and July 2025, a total of 10 patients with native (after balloon valvuloplasty) or surgically patched RVOTs underwent TPVR using the Alterra Pre-Stent and Edwards SAPIEN 3 valve. Patient selection, procedural data, and early clinical outcomes were reviewed.

**Results:**

All 10 patients (mean age: 30 ± 7.1 years; range: 15-40 years) underwent successful implantation. The mean weight was 66.4 ± 22.7 kg (range: 35-103 kg), and 30% (n = 3) were male. Procedural success was 100%, with accurate device positioning and no significant residual gradient or pulmonary regurgitation. No major complications—including valve embolization, coronary compression, or surgical conversion—occurred. At a median follow-up of 31 days (range: 10-43 days), all patients remained clinically stable with improved functional status.

**Conclusions:**

This is the first reported experience of the Alterra Adaptive Pre-Stent in the Middle East. Early results demonstrate that the device is safe, technically feasible, and effective in selected patients with complex RVOT anatomies, potentially broadening TPVR applicability in the region.

## Résumé

**Contexte:**

Le dispositif Alterra Adaptive Pre-Stent, utilisé avec la valve Edwards SAPIEN 3, offre une nouvelle solution d’implantation transcathéter pour les patients présentant une voie de sortie ventriculaire droite (VSVD) fortement dilatée qui ne convient pas à l’implantation transcathéter de valve pulmonaire (ITVP) classique. Aucun rapport ne fait état de l’expérience préalable avec ce dispositif au Moyen-Orient.

**Méthodologie:**

Nous faisons état pour la première fois de l’expérience avec le dispositif Alterra Adaptive Pre-Stent au Moyen-Orient dans un centre de cardiologie très achalandé spécialisé dans les cardiopathies congénitales. Nous avons réalisé une analyse rétrospective dans un seul centre, à savoir le Prince Sultan Cardiac Center (Riyad, Arabie saoudite). Entre avril et juillet 2025, un total de 10 patients présentant une VSVD native (après valvuloplastie au ballonnet) ou corrigée chirurgicalement ont subi une ITVP à l’aide du dispositif Alterra Pre-Stent et de la valve Edwards SAPIEN 3. La sélection des patients, les données relatives à l’intervention et les premiers résultats cliniques ont été examinés.

**Résultats:**

L’implantation a été une réussite chez les 10 patients (âge moyen : 30 ± 7,1 ans; plage de 15 à 40 ans). Le poids moyen était de 66,4 ± 22,7 kg (plage de 35 à 103 kg), et 30 % des patients (n = 3) étaient des hommes. La réussite de l’intervention a été de 100 %; le positionnement du dispositif était précis et aucun gradient résiduel significatif ni aucune régurgitation pulmonaire n’ont été observés. Aucune complication majeure, comme l’embolisation valvulaire, la compression coronaire ou la conversion chirurgicale, n’est survenue. Après un suivi médian de 31 jours (plage de 10 à 43 jours), tous les patients sont restés cliniquement stables avec un statut fonctionnel amélioré.

**Conclusions:**

Il s’agit du premier rapport faisant état de l’expérience avec le dispositif Alterra Adaptive Pre-Stent au Moyen-Orient. Les premiers résultats indiquent que le dispositif est sûr et l’intervention techniquement réalisable et efficace chez certains patients présentant une VSVD anatomiquement complexe, ce qui pourrait élargir l’applicabilité de l’ITVP dans la région.

Pulmonary regurgitation (PR) and progressive right ventricular (RV) dilatation remain common late complications in patients with congenital heart disease, including patients who have undergone surgical reconstruction of the RV outflow tract (RVOT), especially in cases such as tetralogy of Fallot. Left untreated, chronic PR may lead to RV dysfunction, arrhythmias, and heart failure.^1^^,^^2^

Transcatheter pulmonary valve replacement (TPVR) has become an increasingly adopted alternative to surgical intervention, offering reduced procedural risk and faster recovery.^3^ However, balloon-expandable TPVR—using the Melody and SAPIEN valves—is primarily suitable for patients with pre-existing conduits or homografts that provide a defined landing zone.^4^^,^^5^ In contrast, patients with native or patched RVOTs often have large, irregular anatomies, making standard balloon-expandable TPVR less feasible.

To address this gap, the Alterra Adaptive Pre-Stent system (Edwards Lifesciences, Irvine, CA) was developed. This system serves as a conformable platform that reshapes complex RVOTs to accommodate the Edwards SAPIEN 3 valve (Edwards Lifesciences).^6^ Although preliminary studies from the United States and Europe have reported favorable outcomes,^7^^,^^8^ regional data from the Middle East remain lacking.

The present study reports the initial experience with the Alterra Adaptive Pre-Stent system in a high-volume congenital heart center in Saudi Arabia, focusing on procedural success and short-term clinical outcomes.

## Methods

### Patient cohort and selection

This is a retrospective, single-center study conducted at Prince Sultan Cardiac Center (Riyadh, Saudi Arabia). Between April and July 2025, a total of 10 patients with native or patched RVOTs were evaluated and underwent TPVR using the Alterra Adaptive Pre-Stent system in combination with the Edwards SAPIEN 3 transcatheter heart valve.

Inclusion criteria included body weight ≥25 kg, moderate or greater PR on transthoracic echocardiography (TTE), and an RVOT length of ≥35 mm with a proximal and distal landing zone diameter between 27 and 38 mm. Anatomic eligibility was assessed by echocardiography and cardiac computed tomographic (CT) angiography, based on the feasibility criteria described by Shahanavaz et al.^9^ Each case was approved by an internal multidisciplinary review board before inclusion, and patient selection followed the 2020 ESC Guidelines for the management of adult congenital heart disease.^10^^,^^11^ As this represented a novel technology in our center and region, institutional review board approval was sought and obtained before the introduction of the Alterra/Sapien system, in accordance with the local standard protocol of our institution and after approval by the Saudi Food and Drug Authority.

### Procedural details

All procedures were performed under general anesthesia via femoral venous access. The procedural details of Alterra valve implantation had been previously described.^9^ The Alterra Pre-Stent is a self-expanding nitinol frame partially covered with expanded polytetrafluoroethylene, designed to create a stable landing zone for a 29-mm balloon-expandable Edwards SAPIEN 3 valve within a nonconduit (patched RVOT or native RVOT with no prior surgery) or dilated RVOT.

Procedural anticoagulation was achieved with intravenous heparin to maintain activated clotting time >250 milliseconds. As part of our local standard protocol, antibiotics were administered intraprocedurally and continued for at least 16 hours after the procedure. Aspirin 81 mg daily was initiated within 24 hours of valve implantation and continued indefinitely.

### Follow-up assessments

Patients were clinically evaluated postoperatively at a median follow-up of 31 days (range: 10**-**43 days). All patients underwent TTE 1 day after implantation and TTE with 24-hour Holter assessment at 30-day follow-up. Imaging was analyzed by a designated institutional imaging review committee. Health status assessments included functional class evaluation (New York Heart Association [NYHA] classification). All patients will be screened in the electrophysiology (EP) clinic at 90 days after the procedure.

### Outcomes and end points

The primary end point was acute procedural success, defined as a composite of:

(1) successful deployment of a single Alterra Pre-Stent and a single SAPIEN 3 valve in the intended location,
(2) peak-to-peak right ventricle pulmonary artery gradient <20 mm Hg after implantation,
(3) less than moderate PR on postprocedural or discharge TTE, and
(4) freedom from device explant or mortality at 24 hours after implantation.

Secondary outcomes included improvement in PR severity from baseline to early follow-up, and freedom from major adverse events (MACE) including valve embolization, coronary compression, major vascular complications, or need for surgical conversion.

All adverse events were reviewed and adjudicated by a multidisciplinary clinical events review committee.

### Statistical analysis

Descriptive statistics were used to summarize baseline demographics, procedural success, and early outcomes. Continuous variables were presented as mean or median values with range, depending on distribution. Categorical variables were reported as frequencies and percentages. Given the small cohort size, no formal inferential statistics were applied.

## Results

Of the 21 patients screened, 10 met the predefined cardiac CT criteria for Alterra implantation, which included a nonconduit RVOT (patched RVOT or native RVOT without prior surgery), adequate RVOT diameter to accommodate the device, and appropriate proximal and distal landing zones without significant calcification or distortion. All 10 patients subsequently underwent successful Alterra implantation. The mean procedure sheath time was 67.6 ± 14.9 minutes, with a mean fluoroscopy time of 26 ± 7.3 minutes.

Cardiac magnetic resonance imaging volumetric analysis was performed in all 10 patients, and the results are summarized in Table 1. The mean RV end-diastolic volume indexed to body surface area was 139.3 ± 29.4 mL/m^2^ (range: 97.5-188.0 mL/m^2^), whereas the mean RV end-systolic volume indexed to body surface area was 68.7 ± 17.1 mL/m^2^ (range: 45.9-96.9 mL/m^2^). The mean RV ejection fraction was 50.5% ± 2.2% (range: 48%-54%). All patients demonstrated severe PR. In 4 patients, the RV end-diastolic volume indexed to body surface area was approximately double the left ventricular end diastolic volume indexed, which is considered a threshold criterion for pulmonary valve implantation.^11^ These findings highlight the degree of RV dilation and functional burden present in this cohort.

**Table 1:** Standardized patient data

Patient no.	Age (y)/sex	Diagnosis	WT (kg), HT (m), BSA (m^2^)	NYHA before procedure	TV regurgitation before procedure	LVEDV (mL)/LVEDVi (mL/m^2^)	LVESV (mL)/LVESVi (mL/m^2^)	LVEF%	RVEDV (mL)/RVEDVi (mL/m^2^)	RVESV (mL)/RVESVi (mL/m^2^)	RVEF%
1	29/F	PA/VSD/PDA Post right BTT 1995 Post repair with TAP 1997	59.0, 169, 1.66	II	Mild TR	99/59	40/24	59	238/143	117/70	51
2	33/F	TOF S/P repair with TAP 1993	90, 164, 2.07	II	Mild TR	142/69	48/23	66	245.5/122	116/58	52.7
3	37/F	Severe PS Post PS ballooning 1989 Post PDA device closure 1993 Free PR	81.0, 162, 1.91	II	Mild TR	124/65	50/26	59	191/100	98/51	48
4	32/M	Critical PS Post PS ballooning Post valvotomy and PDA ligation 1992 Post TV repair and PA reconstruction with PS resection and PFO closure in 1998	66, 173, 1.78	II	Moderate TR	149/83	67/37	55	304/171	157/88	48
5	15/F	TOF variant S/P repair with TAP age of 1 year (2009)	51.0, 160, 1.51	II	Mild TR	120/79	51/33	58	222/146	108/71	51
6	28/M	TOF Post repair with TAP 1997	44, 162, 142	II	Mild TR	96/67	30/21	68	265/187	137/97	48
7	23/F	TOF Post right BT shunt 2002 Post TAP 2003	35, 147, 1.2	II	No TR	96/80	32/27	67	199/165	103/86	48
8	34/F	TOF Post repair with TAP + PFO closure 1989	87.0, 158, 1.95	II	Trivial TR	125/64.3	51.8/26.5	58	249.5/128	122/62	51
9	32/F	TOF Repair at 4 months (lost to follow-up) Severe PR (exertional dyspnea and palpitations for 10 years)	48, 150, 1.41	III	Moderate TR	93/66	34/24	64	188/133	91/61	54
10	40/M	PA/IVS Post left BT shunt + pulmonary valvotomy 1984 Post pulmonary outflow reconstruction + BT shunt closure and reattaching LPA 1988 Severe PR Post PV ballooning Severely symptomatic	103, 172, 2.22	III-IV	Severe TR	132/59	53/23	59	216/97	101/45	52

BSA, body surface area; BTT, Blalock-Thomas-Taussig; F, female; HT, height; LPA, left pulmonary artery; LVEDV, left ventricular end diastolic volume; LVEDVi, left ventricular end diastolic volume indexed; LVEF, left ventricular ejection fraction; LVESV, left ventricular end systolic volume; LVESVi, left ventricular end systolic volume indexed; M, male; NYHA, New York Heart Association; PA, pulmonary atresia; PDA, patent ductus arteriosus; PFO, patent formaen ovale; PR, pulmonary regurgitation; PS, pulmonary stenosis; PV, pulmonary valve; RVEDV, right ventricular end diastolic volume; RVEDVi, right ventricular end diastolic volume indexed; RVEF, right ventricular ejection fraction; RVESV, right ventricular end systolic volume; RVESVi, right ventricular end systolic volume indexed; S/P, status post; TAP, transannular patch TOF, tetralogy of Fallot; TR, tricuspid regurgitation; TV, tricuspid valve; VSD, ventricular septal defect; WT, weight.

Functionally, our patients were in NYHA class II-IV at baseline. At 1-month follow-up, all patients reported symptomatic improvement, with all transitioning to NYHA class I after the procedure. Preprocedurally, all patients demonstrated free PR, which resolved after valve deployment, with only trivial PR observed in 2 patients at 1-month follow-up. No significant progression of tricuspid regurgitation (TR) was observed; in most patients, TR was either stable or reduced to mild/trivial. One patient had severe TR before valve implantation that decreased to moderate TR 1 month after implantation. Left ventricular ejection fraction (LVEF) remained preserved, with no clinically meaningful changes observed (mean preimplant LVEF: 54.6% and mean postimplant LVEF: 56.0%). The RVOT/pulmonary valve gradient decreased from 22.7 ± 2.2 mm Hg before the procedure to 9.0 ± 3.9 mm Hg after the procedure, representing a mean reduction of 11.1 ± 4.3 mm Hg (*P* < 0.001). Importantly, no pericardial effusion (PE) was detected in any patient during follow-up.

**Figure 1:**
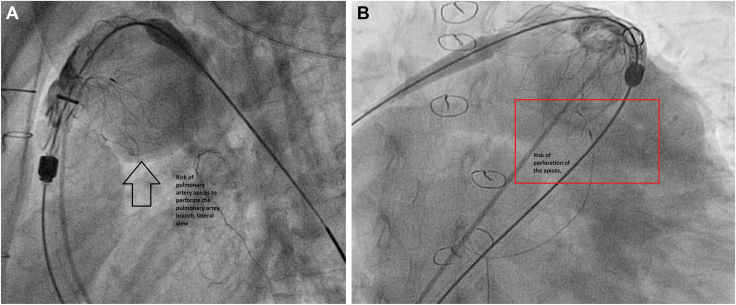
(**A**) Lateral view showing apices of the stent perpendicular to the pulmonary artery while stiff wire in the right pulmonary artery. (**B**) Right anterior obligue (RAO) view showing apices of the stent perpendicular to the pulmonary artery while stiff wire in the right pulmonary artery.

**Figure 2:**
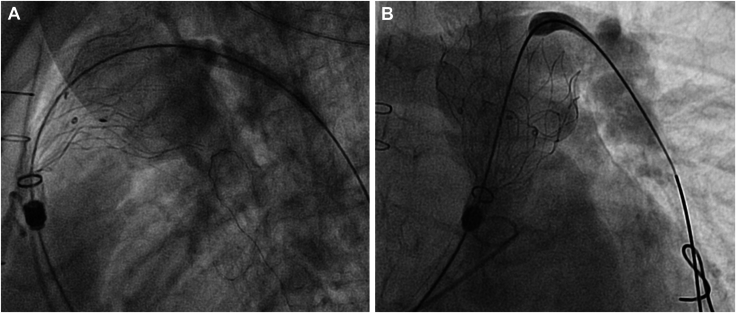
(**A**) Same patient, Lateral fluoroscopy view, with apices coaxial to the pulmonary artery wall, when the stiff wire is repositioned to the left pulmonary artery. (**B**) Same patient, RAO fluoroscopy view, with apices coaxial to the pulmonary artery wall, when the stiff wire is repositioned to the left pulmonary artery.

In all but 2 patients, Alterra Pre-Stent deployment was performed over a stiff wire positioned in the right pulmonary artery (RPA). In one case, although the initial wire position was in the RPA, it was subsequently repositioned to the left pulmonary artery (LPA) for Alterra Pre-Stent deployment. This adjustment, which deviated from preprocedural CT recommendations, was made to achieve improved Alterra Pre-Stent shouldering and alignment. *Shouldering* of the Alterra refers to the positioning in which the device’s distal flare rests along, and conforms to, the angular junction between the main pulmonary artery and the origin of a branch pulmonary artery. In this configuration, the flare’s contour is “shouldered” against the branch take-off, providing stable anchoring without protruding into the branch lumen. Recognizing this relationship on pre- and postimplant imaging is important, as optimal shouldering can enhance device stability, minimize migration risk, and ensure unobstructed flow into both pulmonary artery branches. In this patient, the origin of the left main coronary artery (LMCA) was in close proximity to the anticipated deployment site, necessitating a slightly higher Alterra Pre-Stent position to mitigate coronary compression risk. After successful Alterra Pre-Stent deployment, the wire was repositioned into the RPA to facilitate valve implantation. In another patient, the wire was initially placed in the RPA; however, during Alterra Pre-Stent deployment, after approximately 50% of the prestent was released, the proximal apices were noted to be angulated perpendicularly to the pulmonary artery wall, posing a potential risk of perforation (Fig. 1, Videos 1 and 2 , view video online). The device was consequently recaptured, and the wire was repositioned into the LPA. This adjustment allowed for a more coaxial alignment between the stent and the pulmonary artery, thereby reducing the risk of vascular injury (Fig. 2, Videos 3 and 4 , view video online).

Two patients (20% of the cohort) developed nonsustained ventricular tachycardia (VT) after valve implantation, with preserved cardiac output throughout. In the first case, VT occurred during wire advancement, before stent introduction, and resolved after a single nonsynchronized direct current shock. The patient remained stable for the remainder of the procedure. In the second case, brief runs of VT occurred intraprocedurally and resolved spontaneously. This patient also experienced short runs of nonsustained VT after the procedure. Both patients were maintained on oral β-blockers (metoprolol), with complete resolution of arrhythmias within 48 hours of implantation. β-Blocker therapy was continued after discharge. A 30-day Holter monitor was performed in 5 patients, including both individuals with prior VT, and demonstrated no arrhythmias or recurrent VT episodes. These findings suggest that although ventricular arrhythmias may occur periprocedurally, they can be effectively managed with prompt intervention and short-term β-blocker therapy, without impacting short-term procedural safety.

## Discussion

To our knowledge, this study presents the first reported Middle Eastern experience with the Alterra Adaptive Pre-Stent system. In our series of 10 patients with large, native or patched RVOTs, we achieved 100% procedural success with no MACE. It is important to note that not all patients were suitable candidates for Alterra device implantation because of unfavorable anatomical features—most notably, short and pyramidal RVOTs, type I RVOT,^12^ which may compromise adequate engagement of the system. Such patients were excluded during the initial phase of the study.

Preprocedural assessment is critical for safe and effective stent implantation, allowing early identification of potential complications and planning of optimal wire positioning. In one patient, preprocedural CT suggested the placement of the stiff wire in the RPA; however, intraprocedural fluoroscopy revealed that the distal stent apices were nearly perpendicular to the pulmonary artery wall during deployment (Fig. 1, Videos 1 and 2 , view video online), raising concern for possible vessel injury.

The Alterra Pre-Stent features outwardly flared proximal and distal ends: the proximal flare engages the pulmonary artery above the annulus, and the distal flare anchors within the RVOT.^13^ These flares distribute radial force over a broader surface area to enhance stability, reduce migration risk, and facilitate coaxial valve alignment. In certain anatomies—particularly with acute RVOT**-**PA angulation or reduced vessel compliance—the flares may exert localized pressure on the vessel wall. If combined with suboptimal coaxial alignment or oversizing, this could theoretically increase the risk of intimal injury or, in rare cases, perforation. Reports in the literature describe pulmonary artery wall impingement by stent apices leading to PE. Ligon et al.^13^ documented 6 such cases from multiple centers in which patients developed either immediate or delayed PEs requiring drainage; surgical exploration confirmed protrusion of stent apices through the pulmonary artery wall.^13^ These findings reinforce the importance of ensuring coaxial alignment during deployment. In our patient, malalignment was recognized after approximately 50% of the stent was deployed (Videos 1 and 2 , view video online). The device was recaptured, and—despite initial CT guidance—the stiff wire was repositioned into the LPA. This provided a more favorable coaxial trajectory and mitigated the potential perforation risk (Fig. 2, Videos 3 and 4 , view video online).

In another case, inadequate shouldering of the Alterra valve was observed over the pulmonary artery angle while the stiff wire was positioned in the RPA, leading to lower implantation position and embedment into the RVOT rather than engagement of the stent on the pulmonary artery shoulder. Consequently, the wire was repositioned into the LPA to achieve improved shouldering and facilitate more controlled deployment of the Alterra prestent. This adjustment allowed for a higher stent position, which was necessary given the proximity of the LMCA to the RVOT, thus minimizing the risk of coronary compression. After successful prestent deployment, the wire was repositioned back into the RPA for valve implantation to prevent engagement of the device by the nose cone during advancement of the valve system or retrieval of the system.

Although earlier reports have described Alterra valve anchoring using the terms "engagement" and "embedment," our experience has led us to redefine this concept with greater specificity.^9^ Type I anchoring involves the direct engagement of the self-expanding frame with the pulmonary artery wall, using a “shouldering” technique between the branch pulmonary arteries and the main pulmonary artery. We favored this technique in most of our patients, as it provided superior stent stability and minimized the risk of stent migration or embolization (Video 5 , view video online). Type II anchoring occurs when the Alterra Adaptive Pre-Stent serves as a docking station within the RVOT, allowing the transcatheter valve to be securely implanted within the prestent. This approach is typically used in cases where a prominent septum between the branch pulmonary arteries prevents adequate shouldering, but it may result in a slightly lower stent position relative to the bifurcation (Video 6 , view video online). This explains why we repositioned the wire, to ensure that the stent deployment achieved type I rather than type II deployment. In our cohort, all but one of our cohort had type I anchoring. Further studies are warranted to better characterize and evaluate both anchoring types. This distinction may serve as a foundation for future research aimed at assessing long-term outcomes and complications associated with each anchoring strategy.

Coronary artery and aortic root compression testing plays a critical role in procedural planning. Baseline computed tomographic angiography was used to assess for coronary anomalies and to evaluate the spatial relationship between the coronary arteries and the RVOT. Given the anatomical variability and the differing engagement positions of the prestent within the RVOT, this remains an important consideration. In cases where the waist of the prestent may abut or exert pressure on adjacent structures, particularly the coronary arteries or aortic root, intraprocedural coronary compression testing can provide valuable safety confirmation before final deployment. One patient in our cohort had the LMCA close to the landing zone in the RVOT, necessitating higher deployment to avoid any compression. No electrocardiogram changes, signs of myocardial infarction, or low cardiac output was noted during deployment of the stent in this patient; therefore, we did not perform any coronary angiography after implantation.

Two of our patients had nonsustained VT requiring medical treatment, which resolved within 48 hours of implantation. Arrhythmias, particularly nonsustained VT, have been reported after Alterra valve implantation. In a multicenter pivotal study, early arrhythmic events occurred in approximately 34% of patients within 24 hours after the procedure, with most resolving with medical therapy.^6^ The valve’s self-expanding frame and its proximity to the RV myocardium may contribute to arrhythmogenic potential. In addition, a recent analysis indicated that clinically significant ventricular arrhythmias were associated with larger frame perimeters and cross-sectional areas in proximity to the RV cavity.^14^ Although a routine preimplantation EP study is not currently standard, it may be prudent in patients with known arrhythmia history or substrate abnormalities. The EP study may help risk-stratify patients and guide procedural planning. Further studies are needed to define standardized arrhythmia surveillance and management strategies.

Other available platforms, such as the Venus P-Valve (MedTech, Hangzhou, China) and the Harmony TPV 22/25 (Medtronic, Dublin, Irleand)), represent valuable TPVR options for selected patients with unoperated or surgically repaired RVOT anatomies. However, in our program, the Alterra/SAPIEN 3 combination was preferred for several reasons. First, the Alterra/SAPIEN 3 system is available in a single size and has a shorter frame, enabling implantation in the pulmonary trunk without extending into the ventricular cavity.^15^ This positioning also allows for future valve-in-valve implantation, prolonging freedom from reintervention, as demonstrated in a retrospective European multicenter registry of the SAPIEN 3 valve for TPVR.^15^^,^^16^ Second, the SAPIEN 3 valve allows controlled, single-inflation deployment with precise fluoroscopic markers within the Alterra waist, a technique familiar to operators in our center with prior transcatheter aortic valve implantation/TPVR experience.^9^^,^^17^ This familiarity, combined with the balloon-expandable mechanism, often results in a shorter learning curve and more predictable deployment compared with fully self-expanding valve systems, where gradual release and frame expansion may require multiple adjustments for optimal valve positioning.^18^^,^^19^ In addition, our team already had extensive experience with the SAPIEN platform through established transcatheter aortic valve implantation and TPVR programs, which facilitated procedural planning, device handling, and imaging guidance, further minimizing technical challenges. The Alterra/SAPIEN 3 system is also readily available through institutional procurement channels, supporting efficiency and timely access. Importantly, the choice between these platform systems is not hierarchical but is determined by multimodality imaging assessment, market availability, and operator familiarity, with the Alterra/SAPIEN system filling a crucial niche for patients who would otherwise have no percutaneous option and face higher-risk surgical reintervention.

Given the evolving experience with Alterra valve implantation, and in keeping with the principle of *primum non nocere*, it is prudent for early adopters to initially select anatomically straightforward cases with minimal risk of vascular injury or perforation. Accordingly, not all patients with RVOT are ideal candidates for Alterra implantation, particularly those with complex or atypical anatomy and careful assessment of the anatomy using imaging modalities as cardiac CT and intraprocedural balloon testing is necessary before choosing to deploy the Alterra system.

### Limitations

This study has several important limitations. First, the sample size was small and reflects an early experience limited to 10 patients, which restricts the generalizability of the findings. Second, follow-up was short term, precluding assessment of long-term valve function, structural durability, and late complications such as endocarditis or arrhythmia burden.

## Conclusions

This single-center study presents the first documented experience in the Middle East using the Alterra Adaptive Pre-Stent in combination with the Edwards SAPIEN 3 valve for TPVR in patients with native or patched RVOTs. The procedure was technically feasible in all cases, with no intraprocedural mortality or MACE. Our findings underscore the importance of careful patient selection, preprocedural imaging, and intraprocedural strategies—such as wire positioning and coronary proximity assessment—to optimize safety and outcomes. This initial experience supports the Alterra stent as a promising option in anatomically complex RVOTs previously deemed unsuitable for TPVR.

## Supplementary Material

Video 1 Apices of the prestent perpendicular to pulmonary artery wall, with risk of perforation of the pulmonary artery, lateral view.

Video 2 Apices of the prestent perpendicular to pulmonary artery wall, with risk of perforation of the pulmonary artery, posterior-anterior view, with right anterior oblique angulation.

Video 3 Same patient with the wire repositioned to the left pulmonary artery; now the apices are coaxial to the pulmonary artery wall, lateral view.

Video 4 Same patient with the wire repositioned to the left pulmonary artery; now the apices are coaxial to the pulmonary artery wall, posterior-anterior view, with right anterior oblique angulation.

Video 5 Type I anchoring, with shouldering of the prestent over the pulmonary artery angle.

Video 6 Type II anchoring, prestent serves as a docking station within the right ventricular outflow tract. No shouldering of the stent on the pulmonary artery angle and stent lies in lower position.
